# Photoactive ZnO Materials for Solar Light-Induced Cu_x_O-ZnO Catalyst Preparation

**DOI:** 10.3390/ma11112260

**Published:** 2018-11-13

**Authors:** Magdalena Brzezińska, Patricia García-Muñoz, Agnieszka M. Ruppert, Nicolas Keller

**Affiliations:** 1 Institute of General and Ecological Chemistry, Faculty of Chemistry, Lodz University of Technology, 90-924 Łódź, Poland; brzezinskamm@gmail.com (M.B.); agnieszka.ruppert@p.lodz.pl (A.M.R.); 2 Institut de Chimie et Procédés pour l’Energie, l’Environnement et la Santé, CNRS/University of Strasbourg, 67087 Strasbourg, France; garciamunoz@unistra.fr

**Keywords:** ZnO, photo-oxidation, 4-chlorophenol, Cu_x_O-ZnO catalyst, photodeposition

## Abstract

In this work, the solar light-induced redox photoactivity of ZnO semiconductor material was used to prepare Cu_x_O-ZnO composite catalysts at room temperature with a control of the chemical state of the copper oxide phase. Cu_2_^(I)^O-ZnO and Cu^(II)^O-ZnO composite catalysts were prepared by using Cu(acac)_2_ in tetrahydrofuran-water and Cu(NO_3_)_2_ in water as metallic precursor, respectively. Prior to the implementation of the photon-assisted synthesis method, the most efficient photoactive ZnO material was selected from among different ZnO materials prepared by the low temperature polyol and precipitation methods with carbonates and carbamates as precipitation agents. The photocatalytic degradation of the 4-chlorophenol compound in water under simulated solar light was taken as a model reaction. The ZnO support materials were characterized by X-ray diffraction (XRD), surface area and porosimetry measurements, thermogravimetric analysis (TGA), scanning electron microscopy (SEM) and transmission electron microscopy (TEM), and the synthesis method strongly influenced their photoactivity in terms of 4-chlorophenol degradation and of total organic carbon removal. The most photoactive ZnO material was prepared by precipitation with carbonates and calcined at 300 °C, benefitting from a high specific surface area and a small mean crystallite size for achieving a complete 4-chlorophenol mineralization within 70 min of reaction, with minimum Zn^2+^ released to the solution. Besides thermal catalysis applications, this work has opened a new route for the facile synthesis of Cu_2_O-ZnO heterojunction photocatalysts that could take place under solar light of the heterojunction built between the *p*-type semi-conductor Cu_2_O with direct visible light band gap and the ZnO semiconductor phase.

## 1. Introduction

Zinc oxide (ZnO) nanostructures are materials with potential applications in many fields of nanotechnology, due to the large variety of nanometric structures or architectures that can be synthesized [1]. Besides numerous applications in optoelectronics, electronics, laser technology, converters and energy generators or as gas sensors [1,2,3,4,5,6], zinc oxide (ZnO) nanostructures have received special attention for being used directly as (photo) catalyst or as catalyst support.

Indeed, ZnO is a II–VI compound semi-conductor that has emerged as a promising candidate for being used as heterogeneous photocatalyst under near-UV irradiation in environmental applications, such as the removal of a large range of organic and inorganic contaminants from environmental water and wastewater, including some of the most toxic and refractory molecules in water like pesticides, herbicides, and dyes [7]. It is characterized by a direct wide band gap close to that of anatase TiO_2_ (3.2–3.37 eV), a suitable location of both conduction and valence bands, strong oxidation ability, low cost, abundance and a large exciton binding energy of 60 meV at room temperature [1,8,9,10,11] so that exciton emission processes can persist at or even above room temperature. It has already been reported that the electron lifetime can be significantly higher and that the recombination rate can be lower in ZnO in comparison to TiO_2_, making ZnO attractive and worth investigating for photocatalytic applications [12]. Further, the photocatalytic activity of ZnO can be enhanced by designing ZnO-supported metal nanoparticle photocatalysts, with promising results mainly in the case [13] of Au and Ag [14,15,16,17,18,19]. In such a hybrid metal/ZnO nanostructure, the supported nanoparticles are proposed (i) to act as a sink for photoinduced electrons, so that efficient charge separation could be promoted at the semi-conductor/metal interface with efficient interfacial charge transfer, or (ii) to induce plasmonic effects either through the direct injection of hot (excited) electrons from the metal to the conduction band of ZnO thanks to intimate electrochemical contact between the plasmonic particle and the semiconductor, or through a near-field enhancement mechanism with overlap between the plasmon wavelength and the photocatalyst absorption.

ZnO nanostructures are also widely studied as catalyst support in several reactions of high fundamental or applicative interest, such as methanol oxidation [20]; low-temperature methanol synthesis [21,22]; production of hydrogen from methanol by steam reforming, partial oxidation, or a combination thereof [23]; steam reforming of ethanol; glycerol hydrogenolysis [24]; various transesterification reactions [25]; or, selective hydrogenation reactions [26,27,28,29,30]. The active metals or metal oxides have mainly included Cu, Pd, Au, Ag, Co, Ni, Rh, Ir and Pt, and were usually stabilized on the ZnO support by ion exchange, wet or incipient wetness impregnation, or (co)-precipitation [31].

The high interest of using zinc oxide as a support results from its valuable physico-chemical properties. Hydrogenation reactions can benefit from strong metal-support interactions and from the formation of alloys with the supported metals in reductive atmosphere. This behavior is responsible for the modification of the electronic properties of the supported active metals, which in turn results in significant activity and selectivity enhancements in many catalytic reactions [32]. Activity improvement can be also associated with the existence of a hydrogen spillover phenomena taking place in the case of zinc oxide-supported metal catalysts [33,34].

The most-used methods for preparing supported metal catalysts implement consecutive operations, beginning with the adsorption of the metal precursor on the support. The reduction step consists mostly of a thermal treatment under hydrogen, or in a chemical reduction in a solvent with, for example, hydrazine or sodium borohydrate reactants. The catalysts can suffer from heterogeneous supported nanoparticle size distributions, from unwanted temperature-activated reactions between the support and the metal precursor, and from limitations in terms of metal contents.

It has been demonstrated that the synthesis method is playing a key role in the preparation of ZnO materials with varied bulk and surface physico-chemical properties, and many chemical methods have been reported for synthesizing ZnO nanostructures, including notably mechanico-chemical processes; precipitation; sol-gel, solvothermal and hydrothermal methods; methods using an emulsion or microemulsion environment; sonochemical; or, microwave-based methods [4,5,6,11].

Therefore, the aim of this article is two-fold. First, it aims at selecting an appropriate preparation method for synthesizing photoactive ZnO under solar light. This has been performed by studying the influence of the synthesis method on ZnO photoactivity, taking the photocatalytic degradation of 4-chlorophenol in water under solar light as a model reaction. Further, the solar light-induced redox photoactivity developed by ZnO has been used for room-temperature preparation of Cu_x_O-ZnO catalysts that are of interest in fundamental and applicative reactions, and that do not require the use of any thermal treatment, or of any gaseous or liquid reductant for controlling the Cu chemical state.

## 2. Materials and Methods

### 2.1. Synthesis of ZnO Materials

#### 2.1.1. Polyol Method

In the polyol synthesis, 1.5 g of zinc(II) acetate dihydrate (Zn(OAc)_2_·2H_2_O, Sigma-Aldrich, Saint Louis, MO, USA, ACS reagent, ≥98%) was introduced into 50 mL of propane-1,3-diol solvent (C_3_H_8_O_2_, Sigma-Aldrich, 98%). The obtained mixture was kept under continuous stirring and under reflux at 160 °C for 15 min or 60 min. Afterwards, the precipitate obtained was cooled, centrifuged for 20 min at 3500 rpm, and finally washed and filtrated under vacuum several times with absolute ethanol (Sigma-Aldrich). The synthesized powder was dried at 100 °C for 12 h. The samples obtained were labelled as ZnO-P-15 and ZnO-P-60 according to the reflux duration.

#### 2.1.2. Precipitation with Na_2_CO_3_

In the precipitation synthesis method, 1.75 g of zinc(II) acetate dihydrate and 0.84 g of sodium carbonate (Na_2_CO_3_, Sigma-Aldrich, 99.5%) were dissolved under stirring in 50 mL of distilled water, respectively. Both aqueous solutions were mixed, and the obtained precipitate was aged at room temperature in the mother liquor for 24 h under continuous stirring. The suspension was further centrifuged for 30 min at 3500 rpm, and finally washed and filtrated under vacuum with distilled water. The resulting powder was dried at 100 °C for 12 h and subsequently calcined at a temperature of 300 °C to 500 °C for 2 h with a 10 °C/min heating rate, leading to the ZnO-C material series.

#### 2.1.3. Precipitation with NH_2_CO_2_NH_4_

Quantities of 10.98 g of zinc(II) acetate dihydrate and 4.29 g of ammonium carbamate (NH_2_CO_2_NH_4_, Sigma Aldrich, 99%) were dissolved under stirring in 50 mL of distilled water. Both aqueous solutions were mixed, and the obtained precipitate was aged at room temperature in the mother liquor for 30 min under continuous stirring. The suspension was further centrifuged for 30 min at 3500 rpm, and finally washed and filtrated under vacuum with distilled water. The resulting powder was dried at 100 °C for 12 h and subsequently calcined at a temperature of 400 °C to 500 °C for 2 h with a 10 °C/min heating rate, leading, e.g., to the ZnO-c material series.

### 2.2. Photon-Assisted Preparation of Cu-ZnO Catalysts

The photon-assisted preparation of Cu-ZnO catalysts was performed by irradiating with solar light a suspension of the ZnO support containing Cu(acac)_2_ or Cu(NO_3_)_2_ as metallic precursors. The irradiation was provided by an Atlas Suntest XLS+ reaction chamber equipped with a Xenon arc lamp adjusted to a 500 W/m^2^ irradiance (with a 30 W/m^2^ UV-A content) (320–800 nm wavelength range, ICH Q1B guidelines). The amount of copper precursor used was adjusted to target a Cu content of 10 wt % on the ZnO support.

#### 2.2.1. Cu(acac)_2_ in THF-H_2_O Solvent

The dissolution of 20.8 mg of copper(II) acetylacetonate (Sigma-Aldrich, 97%) was achieved for 30 min at 60 °C under stirring in tetrahydrofuran (THF, Sigma-Aldrich, 99%)-water mixture with a 2:9 (*v*/*v*) ratio, before 45 mg of the ZnO support was dispersed under stirring in 100 mL of copper solution in a beaker-type glass reactor at a 0.21 g/L concentration. The suspension was first stirred in the dark for 0.5 h at 60 °C for establishing the adsorption/desorption equilibrium, before the photon-assisted synthesis was performed under stirring at 60 °C under solar light for 90 min. The synthesis was monitored by UV-vis spectrophotometry by following the disappearance of the absorption peak at λ = 245 nm. The samples were washed and filtrated under vacuum several times with distilled water, and finally dried at 100 °C for 1 h [13].

#### 2.2.2. Cu(NO_3_)_2_ in H_2_O Solvent

The protocol was similar to that followed with Cu(acac)_2_. In this case, the dissolution of 38 mg of Cu(II) nitrate trihydrate (Sigma-Aldrich, p.a) was performed in water at room temperature, before 90 mg of ZnO was dispersed under stirring in 100 mL of copper solution in a beaker-type glass reactor at a 0.38 g/L concentration. Prior to irradiation, the suspension was stirred in the dark for 30 min at 60 °C to ensure the establishment of the adsorption/desorption equilibrium, before the photon-assisted synthesis was performed under stirring under solar light for 2 h. The synthesis was monitored by UV-vis spectrophotometry by following the disappearance of the absorption peak at λ = 800 nm. The samples were washed and filtrated under vacuum several times with distilled water, and finally dried at 100 °C for 1 h.

### 2.3. Characterization Techniques

The crystallographic structure of the powders was characterized by X-ray diffraction patterns (XRD) recorded on a D8 Advance Bruker diffractometer in a θ/θ mode and using the K_α1_ radiation of a Cu anticathode (λ = 1.5406 Å).

The surface area measurements were carried out on a Micrometrics Tristar 3000 using N_2_ as adsorbent at −196 °C with a prior outgassing at 100 °C overnight in order to desorb the impurities or moisture. The Brunauer-Emmett-Teller (BET) specific surface area was calculated from the N_2_ adsorption isotherm.

Scanning electron microscopy (SEM) was performed in secondary electron mode on a JSM-6700 F FEG microscope (JEOL Ltd., Tokyo, Japan).

Transmission electron microscopy (TEM) was performed using a JEOL 2100F (JEOL Ltd., Tokyo, Japan) with a point resolution of 0.2 nm. A Mo grid was used for performing Energy-dispersive X-ray spectroscopy (EDS) analysis of Cu-ZnO samples. The interplanar spacings were calculated using ImageJ software (National Institutes of Health, Bethesda, MD, USA).

Thermogravimetric analysis (TGA) was carried out with a 20% (*v*/*v*) O_2_/N_2_ mixture at a flow rate of 40 mL/min at a heating rate of 10 °C/min from 25 °C to 600 °C using a Q 5000 thermoanalyzer from TA instrument (New Castle, DE, USA).

The copper content in the catalysts was determined by chemical analysis after a microwave-assisted acidic dissolution in aqua regia at 185 °C under autogenic pressure. Inductively coupled plasma optical emission spectroscopy (ICP-OES) was carried out on an Optima 7000 DV spectrometer (Perkin Elmer, Waltham, MA, USA) at the Analysis Platform of IPHC-Strasbourg, France.

### 2.4. Evaluation of the Photocatalytic Efficiency

The experiments were carried out in an Atlas Suntest XLS+ reaction chamber equipped with a Xenon arc lamp adjusted to a 500 W/m^2^ irradiance (with a 30 W/m^2^ UV-A content) (320–800 nm wavelength range, ICH Q1B guidelines). The tests were performed with a reactor volume of 500 mL and a 4 Cl-phenol concentration of 25 mg L^−1^, corresponding to a carbon load of 14.8 ppm. The ZnO catalyst load was 1 g L^−1^. At each time interval, 20 mL of solution was sampled and then filtered through 0.20 μm porosity filter to remove the photocatalyst powder, if any, before the concentration of 4-chlorophenol was determined by UV-visible spectrophotometry (Cary 100 scan) by monitoring the disappearance of the main absorption peak at λ = 224 nm, and total organic carbon (TOC) was measured with a TOC-L analyzer (Shimadzu, Kyoto, Japan) to determine the organic carbon load.

A procedure was established for photocatalytically cleaning the ZnO materials under UV-A or solar light prior to the chlorophenol removal, consisting in the continuous stirring of the as-synthesized ZnO catalyst in ultrapure Milli-Q water under either simulated solar light for 2 h or UV-A light for 16 h. UV-A light was provided by blacklight lamps (PL-L 24W/10/4P, Philips, Amsterdam, The Netherlands) emitting at 365 nm with an irradiance of 60 W/m^2^, while the UV-A fraction of the simulated solar light corresponded to a 30 W/m^2^ irradiance, as measured using a wide-band RPS900-W rapid portable spectroradiometer from International Light Technology (ILS, Peabody, MA, USA).

## 3. Results

### 3.1. Characterization of ZnO Materials

**Figure 1:**
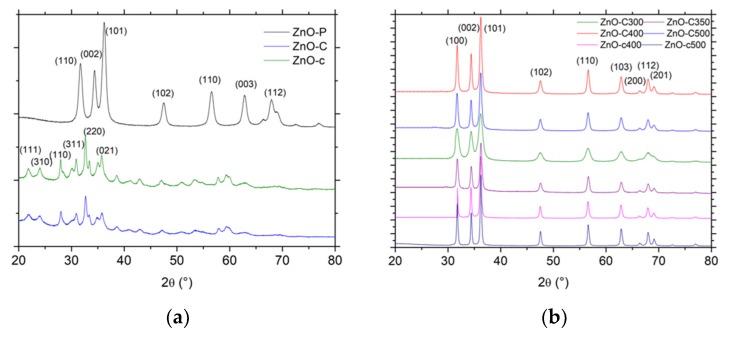
X-ray diffraction patterns (XRD) patterns of (**a**) the dried materials and (**b**) the calcined materials. JCPDS card No. 00-036-1451 and No. 19-1458 for ZnO and the zinc carbonate hydroxide phases, respectively.

The main physico-chemical properties of the ZnO materials are reported in Table 1. Figure 1a,b shows the XRD patterns of the dried ZnO precursors and of the final ZnO materials synthesized via the polyol and the precipitation routes.

In the case of the polyol method, the XRD pattern shown in Figure 1a displayed the diffraction peaks characteristic of ZnO crystallized in the hexagonal wurtzite structure and corresponding to the diffraction of the (100), (002), (101), (102), (110), and (103) planes for the most intense peaks (P63mc space group, JCPDS 00-036-1451) [5,14,15,16]. As reported for the polyol synthesis, the ZnO material had a good crystallinity without any post-synthesis calcination treatment.

By contrast, the dried material obtained in the case of both precipitation methods consisted of a Zn_5_(CO_3_)_2_(OH)_6_ zinc carbonate hydroxide, or hydrozincite phase, in the monoclinic structure [17] with the diffraction of the (111), (310), (100), (311), (220) and (021) planes for the most intense peaks (JCPDS 19-1458). Han et al. [18] have proposed that the reactions during the formation of Zn_5_(CO_3_)_2_(OH)_6_ are likely as follows:

(1) $$\;\mathrm{Zn}{\left({\mathrm{CH}}_{3}\mathrm{COO}\right)}_{2}\to {\mathrm{Zn}}^{2+}+2{\left[{\mathrm{CH}}_{3}\mathrm{COO}\right]}^{-}\;$$

(2) $$\;{\left({\mathrm{CH}}_{2}\right)}_{6}N_{4}+10H_{2}O\to 6\mathrm{HCHO}+4{\mathrm{OH}}^{-}+4{\left[{\mathrm{NH}}_{4}\right]}^{+}\;$$

(3) $$\;\mathrm{HCHO}+O_{2}+2{\mathrm{OH}}^{-}\to {\mathrm{CO}}_{3}^{2-}+2H_{2}O\;$$

(4) $$\;5{\mathrm{Zn}}^{2+}+2{\mathrm{CO}}_{3}^{2-}+6{\mathrm{OH}}^{-}\to {\mathrm{Zn}}_{2}{\left({\mathrm{CO}}_{3}\right)}_{2}{\left(\mathrm{OH}\right)}_{6}\;$$

**Figure 2:**
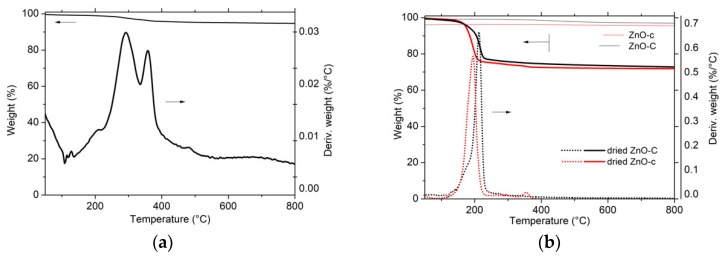
Thermogravimetric analysis (TGA) of the materials after the drying step synthesized by (**a**) the polyol method; (**b**) the carbonate-derived precipitation method and the carbamate-derived precipitation method. The weight loss observed with the ZnO-C and ZnO-c materials after calcination at 300 °C and 400 °C, respectively, was reported.

The zinc carbonate hydroxide phase being thermally stable below 200 °C, its decomposition into ZnO was evidenced by XRD characterization and TGA analysis in Figure 1b and Figure 2, respectively. After calcination of the zinc carbonate hydroxide at a temperature higher than 300 °C, the XRD pattern exhibited the diffraction peaks corresponding to hexagonal ZnO, while, according to the weight loss of 28 ± 1% observed in TGA, the decomposition process can be described as follows, corresponding to a theoretical weight loss of 28.4%:

(5) $$\;{\mathrm{Zn}}_{2}{\left({\mathrm{CO}}_{3}\right)}_{2}{\left(\mathrm{OH}\right)}_{6}\to 5\mathrm{ZnO}+2{\mathrm{CO}}_{2}+3H_{2}O\;$$

The absence of any weight loss during the TGA of the calcined ZnO materials confirmed the efficiency of the thermal decomposition process. By contrast, a very small weight loss of 6% was observed with ZnO prepared via the polyol method, suggesting the residual presence of propane 1-3 diol solvent or acetate species [19].

In agreement with the previous works of Fkiri et al. [14], the ZnO materials synthesized via the polyol method displayed a good crystallinity without any post-synthesis heat treatment, and exhibited the smallest average crystallite size of 7 nm and 11 nm, for an aging duration of 15 min and 60 min, respectively. The ZnO materials prepared via both precipitation routes had a larger mean crystallite size, as a result of the necessary calcination treatment at temperatures ranging from 300 °C to 500 °C. The use of carbonates as precipitating agent allowed maintaining a smaller mean crystallite size in the 10–20 nm range, while the mean crystallite size strongly increased to 28–30 nm in the case of the carbamate agent. For a similar calcination temperature, ZnO prepared with carbamates displayed a larger crystallite size than its carbonate counterpart, e.g., 28 nm vs. 20 nm at a temperature of 400 °C.

**Figure 3:**
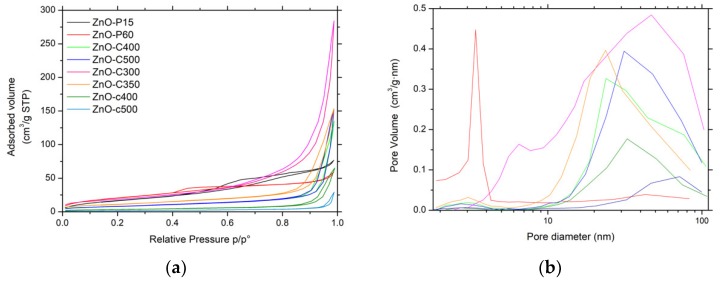
(**a**) N_2_ adsorption-desorption isotherms and (**b**) pore size distributions of ZnO materials.

Figure 3a shows the N_2_ adsorption–desorption isotherms of the ZnO materials. The ZnO-P materials displayed IV-type isotherms, characteristic of mainly mesoporous solids with a mainly H2-type hysteresis corresponding to interconnected mesopores with non-uniform shape or size [14]. For ZnO obtained by precipitation, the isotherms were found to be preferentially of type II with H3-type hysteresis. This suggested that the porosity mainly resulted from macropores (or large mesopores) and is characteristic of aggregates or agglomerates of nanoparticles forming slit-shaped pores with non-uniform size and/or shape. This was in agreement with the corresponding pore size distributions shown in Figure 3b, which clearly evidenced the influence of the synthesis method on the mean pore size.

The specific surface area of the ZnO materials ranged from 6 m^2^/g to 78 m^2^/g without any microporous contribution. Among the different materials, the ZnO-P and ZnO-C-300 samples had the highest specific surface areas, within the 66–78 m^2^/g range, in agreement with their smallest mean crystallite sizes of 7–11 nm. The increase in the calcination temperature for carbonate-derived ZnO materials from 300 °C to 500 °C led to a progressive decrease in the surface area from 68 m^2^/g to 29 m^2^/g, as a result of the slight increase in the mean crystallite size. It is worth noting that the carbamate-derived ZnO samples calcined at 400 °C and 500 °C displayed a lower surface area than their carbonate-derived counterparts, at 12 m^2^/g and 6 m^2^/g respectively, in agreement with a larger mean crystallite size.

The synthesis method had no influence on the band gap of the ZnO materials, graphically estimated at 3.1 eV via the Tauc plot derived from the UV–vis diffuse reflectance analysis, in agreement with the literature (Figure S1b) [14,35]. Therefore, the ZnO materials will be activated by incident photons with wavelengths within the UV-A range, whether the photocatalytic tests are performed under solar light or pure UV-A light.

**Figure 4:**
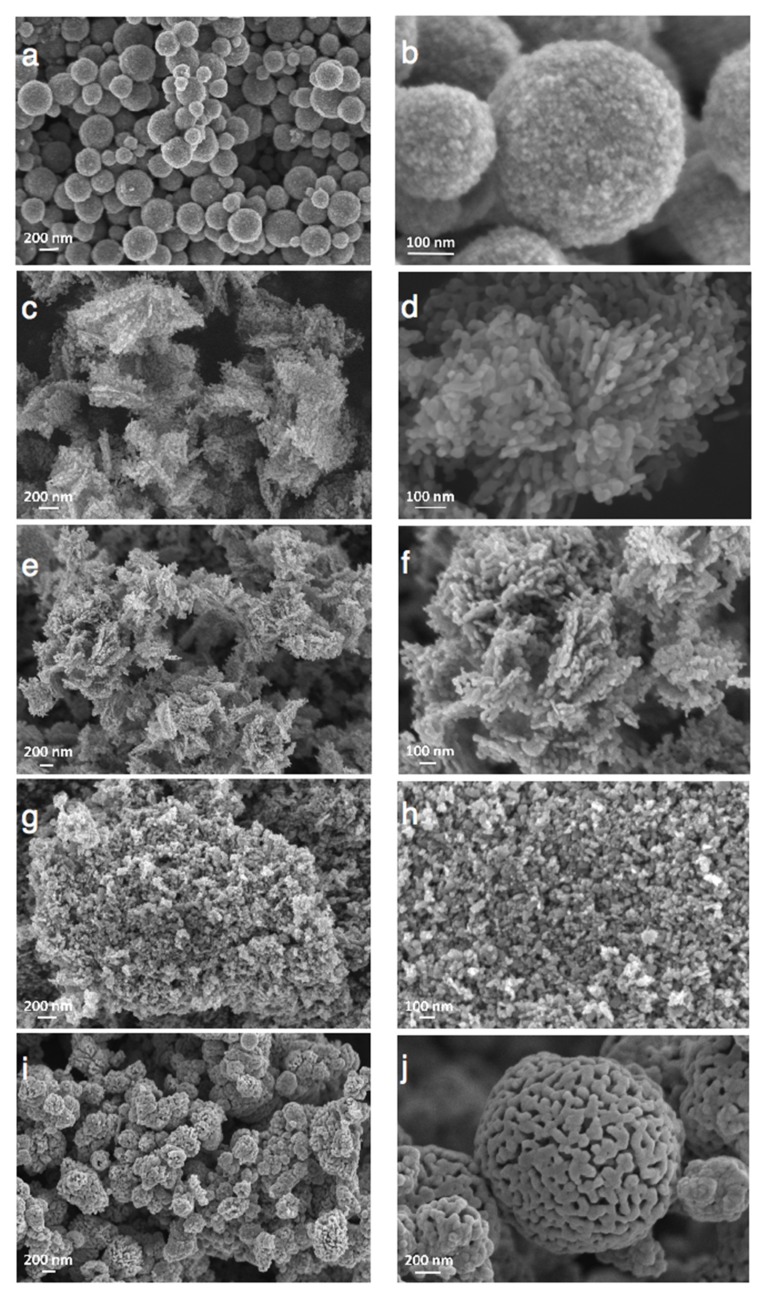
Scanning electron microscopy (SEM) of ZnO materials synthesized through (**a**,**b**) the polyol method (ZnO-P-60); the precipitation with carbonates: (**c**,**d**) ZnO-C300; (**e**,**f**) ZnO-C400; (**g**,**h**) ZnO-C500; and the precipitation with carbamates: (**i**) ZnO-c400 and (**j**) ZnO-c500.

**Figure 5:**
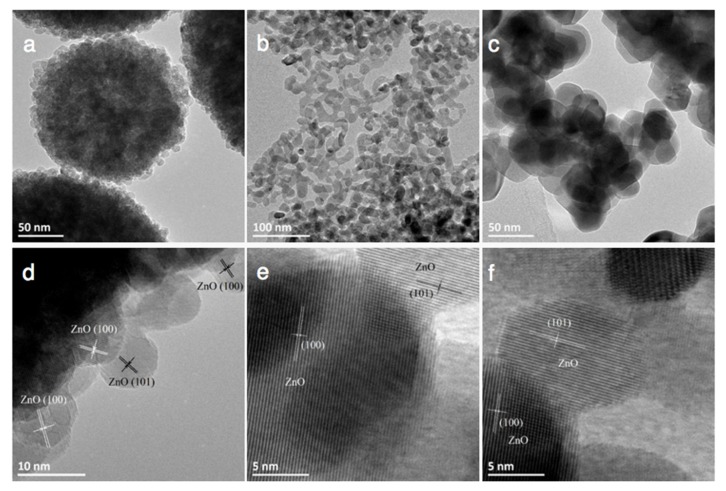
Transmission electron microscopy (TEM) images of ZnO materials synthesized through (**a**,**d**) the polyol method (ZnO-P-60), (**b**,**e**) the precipitation with carbonates (ZnO-C300) and (**c**,**f**) the precipitation with carbamates (ZnO-c400), evidencing the general morphology of the materials and interplanar spacings of 0.28 nm and 0.25 nm consistent with the (100) and (101) planes of hexagonal wurtzite ZnO crystallites.

Figure 4 depicts a selection of scanning electron microscopy (SEM) images of the ZnO materials and evidences that the synthesis method and the nature of the precipitating agent strongly influenced the morphology of the ZnO material. ZnO from polyol synthesis was composed of (100–200 nm) large spherical aggregates of small-size ZnO particles with an average crystallite size around 10 nm, in agreement with that derived from the XRD patterns. The aging duration had no influence on the general morphology of the ZnO-P materials. The interplanar spacing of 0.28 and 0.25 nm shown in Figure 5 was consistent with the (100) and (101) plane of hexagonal (wurtzite) ZnO crystallites.

Transmission electron microscopy (TEM) images of ZnO synthesized via the different methods are shown in Figure 5. Irrespective of the synthesis method, they evidenced interplanar spacings of 0.28 nm and 0.25 nm consistent with the (100) and (101) planes of the hexagonal wurtzite ZnO phase (JCPDS No. 00-036-1451) and confirmed the general morphology of the wurtzite ZnO materials previously observed in SEM images, with crystallite sizes in agreement with those derived from the XRD patterns.

### 3.2. Photocatalytic Activity of ZnO

#### 3.2.1. Influence of a Photocatalytic Pre-Cleaning Step

**Figure 6:**
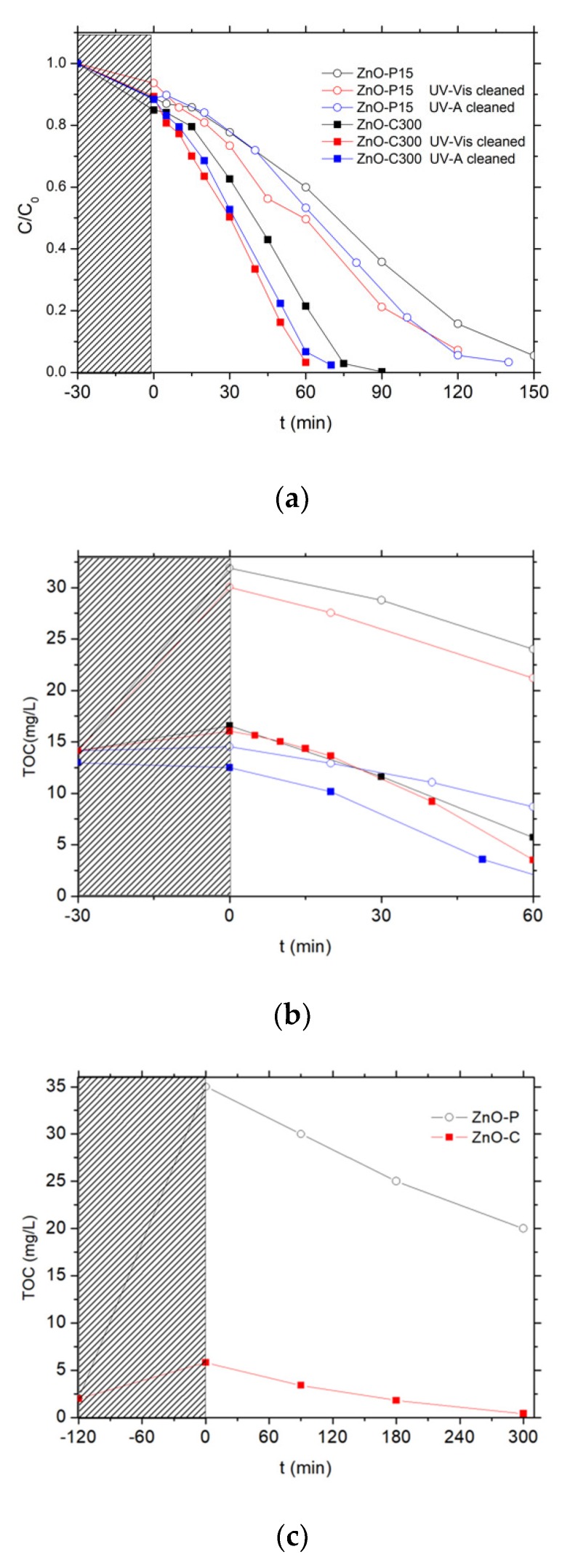
Influence of the photocatalytic pre-cleaning step on (**a**) the relative 4-Cl-phenol concentration and (**b**) the total organic carbon (TOC) concentration evolution upon photocatalysis with ZnO materials. (**c**) Evolution of the TOC concentration during the photocatalytic pre-cleaning step of the as-synthesized ZnO-P and ZnO-C materials under UV-A light in ultrapure Milli-Q water.

The photocatalytic activity of the as-synthesized ZnO materials was first evaluated in water under solar light using 4-Cl-phenol as test molecule. Two catalysts prepared via the polyol method and the carbonate-through precipitation method were selected for studying the influence of a photocatalytic pre-cleaning step, i.e., the dried ZnO-P-15 (sample not subjected to any calcination treatment) and the calcined ZnO-C-300 sample, respectively (Figure 6).

First, no detectable degradation of chlorophenol was observed for 3 h under solar light, demonstrating that the chlorophenol photolysis can be disregarded, as evidenced also by Eslami et al. and Gaya et al. [36,37].

As depicted in Figure 6a, the pollutant concentration evolution with the time under irradiation showed that both ZnO catalysts were able to oxidize the phenolic compound until its complete degradation, achieved within 90 min and 150 min of reaction on ZnO-P-15 and ZnO-C-300. By contrast, a TOC release to the aqueous solution was evidenced during the adsorption period in the dark for both uncleaned ZnO catalysts, before the TOC removal was observed with a kinetic rate constant k’_TOC_ of 0.13 ppm/min and 0.18 ppm/min for ZnO-P-15 and ZnO-C-300 catalysts, respectively (Figure 6b). This TOC release was notably very pronounced in the case of ZnO synthesized via the polyol route, for which no thermal treatment was applied, with a TOC overshoot of 15 ppm at the beginning of the reaction attributed to the release to water of some organic residues from the synthesis, while only 4 ppm of extra TOC was released with the calcined ZnO-C-300 catalyst. This difference in terms of TOC release was in agreement with the higher weight loss recorded on the ZnO-P material and that resulted from the combustion of carbon residues from the synthesis, in comparison to that observed on the calcined ZnO materials.

The catalyst behavior was strongly affected by applying a photocatalytic cleaning step prior to the chlorophenol removal. This cleaning step consisted in submitting an aqueous suspension of the as-synthesized ZnO catalyst either to simulated solar light (2 h, 25 W/m^2^ UV-A irradiance) or to UV-A light (16 h, 60 W/m^2^ irradiance). Figure 6c shows in the case of UV-A that those carboned species can be mineralized during the photocatalytic pre-cleaning step of the as-synthesized materials. The volcano-like TOC profile observed has been attributed to the release and the subsequent mineralization of carbon-containing residues coming from the precursors used in the ZnO synthesis, and that remained adsorbed at the catalyst surface or trapped in the bulk of the ZnO crystallites. It confirmed that ZnO synthesized via the polyol method contained larger amounts of carboned residues than its counterparts obtained via the precipitation approach. They were potentially blocking the ZnO active sites, being consequently in competition with the chlorophenol pollutant for the oxidative species, or acting as recombination centers in the ZnO crystallite bulk for the photogenerated charge carriers.

Performing the cleaning procedure under solar light or UV-A light allowed the degradation rates obtained with the ZnO materials to be improved, as can be observed in Figure 6a,b. Both ZnO catalysts cleaned under solar light or UV-A displayed a faster disappearance of chlorophenol than their uncleaned counterparts. It should be noted that performing the pre-cleaning step for 2 h with a 25 W/m^2^ UV-A irradiance (i.e., solar light as incident light) already allowed a significant enhancement of the kinetic rate constant for the chlorophenol degradation to be obtained, while by contrast the initial TOC release was only slightly improved. The TOC overshoot was totally suppressed only when the pre-cleaning step was performed under UV-A light for 16 h (60 W/m^2^ irradiance).

#### 3.2.2. Photocatalytic Activity of ZnO Materials

**Figure 7:**
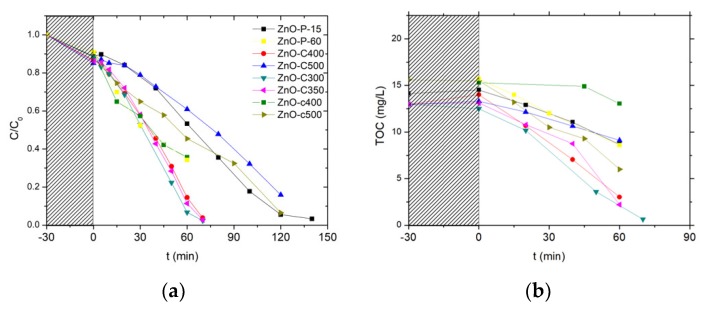
Relative 4-Cl-Phenol concentration (**a**) and TOC concentration (**b**) evolution upon photocatalysis on UV-A light cleaned ZnO materials.

Figure 7 shows the evolution with time under irradiation of the 4-Cl-Phenol and TOC concentrations observed on the different ZnO catalysts after the photocatalytic cleaning step under UV-A light has been applied for 2 h. Table 2 shows the corresponding kinetic rate constants for both the 4-Cl-Phenol and the TOC removal.

Irrespective of the cleaned material tested, no significant TOC release was observed at the beginning of the test, confirming the efficiency of the photocatalytic pre-cleaning step. Globally, the ZnO-C materials prepared by precipitation with carbonates displayed the highest activity for both the removal of 4 Cl-phenol and that of TOC when compared to their counterparts prepared via the polyol method or using the carbamates as precipitation agent. Among the carbonate-through precipitation ZnO series, the highest kinetic rate constants of k’_Phenol_ = 0.21 mg/L/min and k’_TOC_ = 0.18 mg/L/min were obtained with the ZnO-C-300 catalyst, for which the complete mineralization of the pollutant was achieved within 70 min of irradiation. This activity improves the results found in the literature for the removal of 4-chloro-phenol with ZnO catalyst under UVA irradiation [36]. Gaya et al. reached the complete 4-Cl-Phenol depletion in 3 h using 2 g/L of ZnO.

ZnO obtained by the polyol and the carbamate precipitation methods exhibited reduced kinetic constant rates for phenol disappearance and TOC removal, in some cases even two orders of magnitude smaller than that obtained with the most active ZnO. Further, among the ZnO materials prepared with carbonates, ZnO calcined at the highest temperature of 500 °C displayed significantly lower activity compared to that shown by its counterparts calcined in the 300–400 °C range.

We may propose that the ZnO-C-300 photocatalyst takes advantage of a higher specific surface area as well as of a large pore volume and a large mean pore size, that facilitate the access of the 4-chlorophenol reactant and of the reaction intermediates to the ZnO surface sites. By contrast, the high surface area ZnO obtained by the polyol method probably suffered from a very low pore volume and a small mean pore size, while the low surface area of the ZnO-c material prepared with carbamate was assumed to be detrimental to the removal efficiency.

Surface photocorrosion remains one of the main drawbacks of ZnO for use in water treatment. Photocorrosion consists of the dissolution of the ZnO surface with release of Zn^2+^ to the reaction media, so that the catalyst suffers from deactivation with time under irradiation as well as from intrinsic limitation in terms of reusability. The surface corrosion of ZnO is considered to be induced by the photogenerated holes (h^+^) and the overall reaction of the ZnO surface dissolution can be expressed as follows:

(6) $$\;\mathrm{ZnO}+2h^{+}\to {\mathrm{Zn}}_{\mathrm{aqueous}}^{2+}+0.5O_{2}\;$$

According to previous work, the overall surface photocorrosion process consists of two low-rate steps followed by two high-rate steps as follows [38,39]:

(7) $$\;O_{\mathrm{Surface}}^{2-}+h^{+}\to O_{\mathrm{Surface}}^{-}\;$$

(8) $$\;O_{\mathrm{Surface}}^{-}+3O^{2-}+3h^{+}\to 2\left(O-O^{2-}\right)\;$$

(9) $$\;\left(O-O^{2-}\right)+2h^{+}\to O_{2}\;$$

(10) $$\;2{\mathrm{Zn}}^{2+}\to 2{\mathrm{Zn}}_{\mathrm{aqueous}}^{2+}\;$$

**Figure 8:**
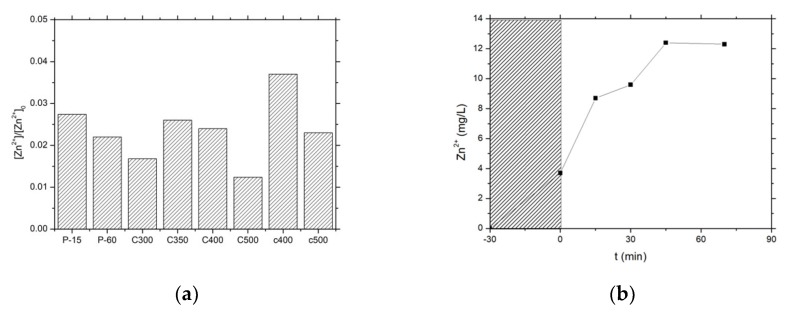
Fraction of Zn^2+^ released into the water (**a**) at the end of the photocatalytic run with ZnO materials and (**b**) as a function of time under irradiation in the case of the ZnO-C-400 catalyst.

Therefore, it was of high interest to evaluate the influence of the synthesis method on the stability of the ZnO photocatalysts in terms of Zn^2+^ release to the media, expressed as the percentage of Zn^2+^ released from the ZnO catalysts into the water (Figure 8). In a first approximation, the materials with a higher activity exhibited a higher stability, with a lower release of Zn^2+^ cation to the solution being observed for the most active catalysts and globally a lower release for the ZnO material series prepared by precipitation with carbonates, e.g., a 0.016 fraction in the case of the most active catalyst ZnO-C-300.

The ZnO-C-500 material displayed better stability than its counterparts calcined at 300 °C, but suffered from lower activity under UV-A light. Therefore, based on the kinetic rate constants derived from both the chlorophenol disappearance and the TOC evolution curves, as well as on the level of Zn^2+^ release to the media, this fast screening allowed selecting the ZnO-C-300 material obtained via the precipitation method with carbonates and calcined at 300 °C, for implementing subsequently the solar light photon-assisted preparation of the Cu-ZnO hybrid catalysts in the next section.

However, we would like to point out that the surface photocorrosion of ZnO materials remained a key issue for using such materials as photocatalysts in water treatment. Indeed, a strong loss of activity was observed when performing sequential runs on the ZnO-C-300 photocatalyst. Using the TOC conversion achieved after 75 min of test as an indicator (i.e., the duration necessary for achieving full TOC conversion in the run#1), Table 3 evidences that the surface photocorrosion was accompanied by an important loss of activity with sequential runs.

### 3.3. Preparation of Cu-ZnO Catalysts by the Photon-Assisted Synthesis Method

**Figure 9:**
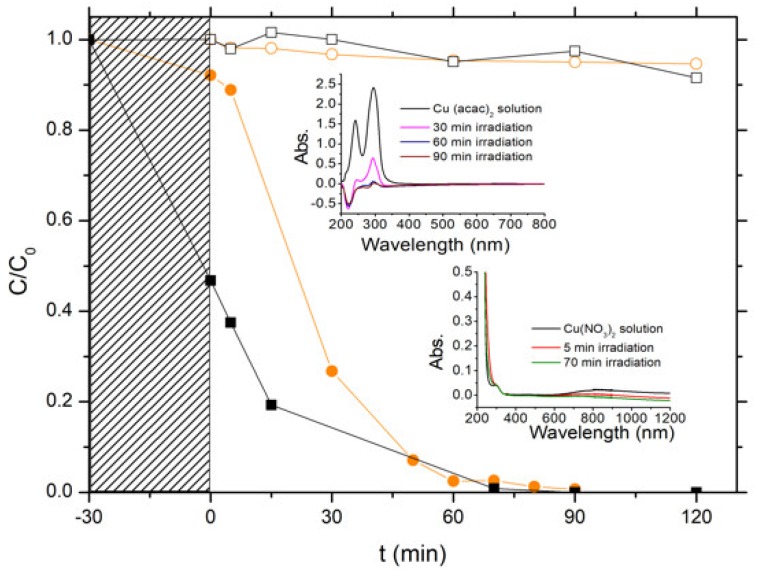
Disappearance curves of (￭) the Cu(NO_3_)_2_ and (●) the Cu(acac)_2_ precursors during the photon-assisted synthesis in the presence of the ZnO-C-300 catalyst. Blank photolysis experiments in the absence of ZnO photocatalyst for (□) Cu(NO_3_)_2_ and (￮) Cu(acac)_2_ precursors. Insert: Time-evolution of the UV-vis absorbance spectra for both precursors (selected analysis times are reported for not overloading the UV-vis absorbance graphs).

The photon-assisted preparation of the Cu-ZnO catalysts was performed using the ZnO-C-300 material as semi-conductor support, since the first part of this work evidenced that it displayed the highest photoactivity in terms of removal of both chlorophenol and TOC, and also showed a high photo-stability in water. The photon-assisted synthesis method takes advantage of the redox photo-activity of the ZnO semiconductor under solar light for allowing the reduction of the metal ions adsorbed at the host surface by the photogenerated electrons from the conduction band of the irradiated semi-conductor. Both Cu(acac)_2_ and Cu(NO_3_)_2_ metallic precursors were used, and Figure 9 depicts the disappearance curves of both precursors during the photon-assisted synthesis in the presence of the ZnO-C-300 catalyst, derived from the time-evolution of the UV-vis absorbance spectra.

First, photolysis of the copper precursors under solar light in our experimental conditions could be disregarded, since no changes of the UV-vis spectra were observed irrespective of the precursor used (not shown). The evolution with time of the C/C_0_ relative concentrations evidenced that the photodeposition occurred on the ZnO support, and further that the copper precursor nature influenced the kinetics of the photodeposition process at the surface of the irradiated ZnO support. However, it has to be noted that time-monitoring of the photo-deposition process was more difficult using the nitrate precursor than its acetylacetonate counterpart. Indeed, the ability of semi-conductor photocatalysis to perform nitrate reduction and nitrate oxidation in water led to the production first of nitrite as initial intermediate (very unstable and easily oxidized back to nitrate) and further higher oxidation state products such as ammonia. The UV-vis signature of those N-compounds is known to overlap around similar absorption wavelengths, so that the photodeposition was monitored in a first approximation by following the disappearance of the low intensity absorption peak at λ = 800 nm related to the Cu^2+^ concentration, which resulted in less accurate time-monitoring. However, ICP-OES analysis revealed that the Cu-ZnO catalyst had a Cu content of 9.7 wt %, in good agreement with the targeted theoretical amount of 10 wt %. By contrast, only a Cu content of 7 wt % was obtained in the case of the Cu(acac)_2_ precursor. This difference was attributed to a possible release of non-steadily anchored Cu species during the washing of the Cu-ZnO materials directly after the photon-assisted synthesis.

### 3.4. Characterization of Cu-ZnO Catalysts

**Figure 10:**
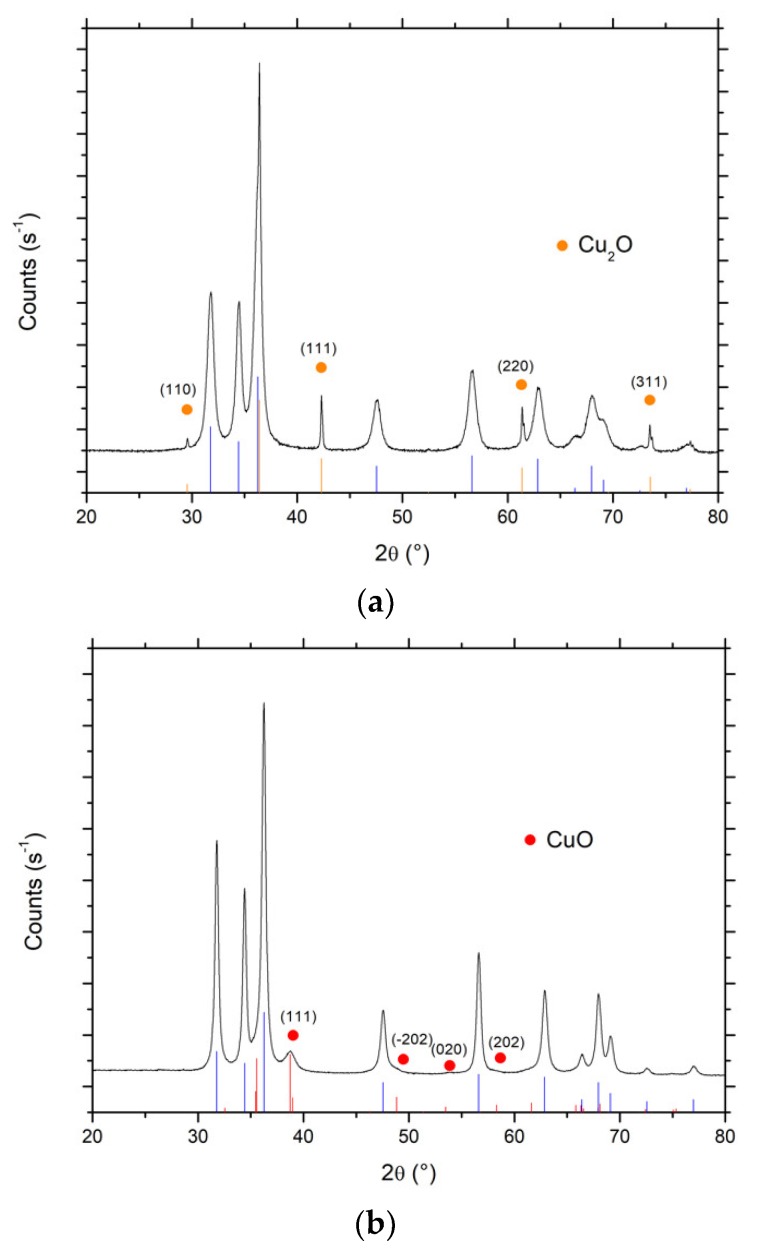
XRD patterns of the Cu_x_O-ZnO materials synthesized by photodeposition using (**a**) Cu(acac)_2_ and (**b**) Cu(NO_3_)_2_ as metallic precursor. Main reflexes from JCPDS cards No 01-089-5895 and 00-005-0667 for CuO and Cu_2_O phases, respectively. The main reflexes from JCPDS card No 00-036-1451 for the ZnO support are shown in blue.

Figure 10 shows the XRD patterns of both Cu-ZnO catalysts. In the case of the Cu(acac)_2_ precursor, besides the diffraction peaks of ZnO crystallized in the hexagonal wurtzite structure, additional diffraction peaks were observed at 29.6°, 42.2°, 61.3° and 73.4°, and attributed to the diffraction of (110), (111), (220) and (311) planes of cubic Cu^(I)^_2_O nanoparticles with an average crystallite size of 65 nm (JCPDS Card 00-005-0667). By contrast, in the case of Cu(NO_3_)_2_ precursor, additional diffraction peaks and shoulders have been recorded at 2θ = 38.8°, 54.0° and 58.0°, and attributed to the diffraction of (111), (020) and (202) planes of monoclinic Cu^(II)^O nanoparticles with an average crystallite size of 10 nm (JCPDS Card 01-089-5895). Interestingly, the choice of the photon-assisted synthesis/deposition parameters allowed selective driving of the oxidation state of the Cu nanoparticles synthesized on the ZnO support towards either cuprous or cupric oxides, so that the prepared catalysts could be considered as Cu^(I)^_2_O-ZnO or Cu^(II)^O-ZnO. This suggests the possibility of controlling the oxidation state of the Cu nanoparticles in the Cu-ZnO while implementing a preparation procedure at room temperature without applying any final thermal treatment.

In the case of the Cu nitrate precursor, the reduction of adsorbed Cu^2+^ ions by the photogenerated electrons would first give Cu^0^, which might be further re-oxidized by the OH radicals formed via the oxidation of adsorbed water by the photogenerated holes from the valence band, or directly by the holes. Cu^0^ was also suggested to undergo reoxidation by O_2_ into Cu^2+^ [40], the Cu^2+^/Cu^0^ couple being then considered to act as an electron mediator from the conduction band to O_2_. In the case of the acetylacetonate precursor, the direct Cu(acac)_2_ reduction into Cu^0^ was reported to be strongly unfavored compared to that of Cu^2+^ [40]. By contrast, the adsorbed acetylacetonate precursor was proposed to be first oxidized by OH radicals formed via the oxidation of adsorbed water by the photogenerated holes from the valence band, or directly by the holes. Subsequently, the ligand oxidation would generate adsorbed Cu^2+^ ions that can be subsequently reduced by the photogenerated electrons.

Further complementary research is being performed for understanding the mechanisms involved in the selective formation of Cu^2+^ or Cu^+^ species at the surface of the ZnO support and for shedding light on the main synthesis parameters that enable driving of the oxidation state of the Cu within the Cu-ZnO material.

It should be noted that performing the photodeposition process on the ZnO support led to an increase in the mean crystallite site of the semi-conductor support from 10 nm to 20 nm in the case of the copper nitrate precursor. By contrast, no significant change was observed in the case of the acetylacetonate precursor, with a mean crystallite size of 13 nm being obtained.

**Figure 11:**
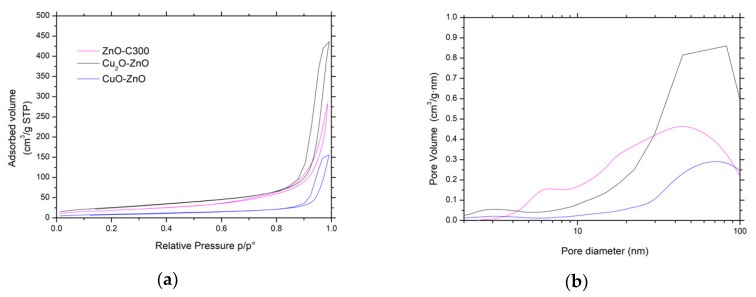
(**a**) N_2_ adsorption-desorption isotherms and (**b**) pore size distributions of both Cu_x_O-ZnO catalysts and bare ZnO material as reference.

**Figure 12:**
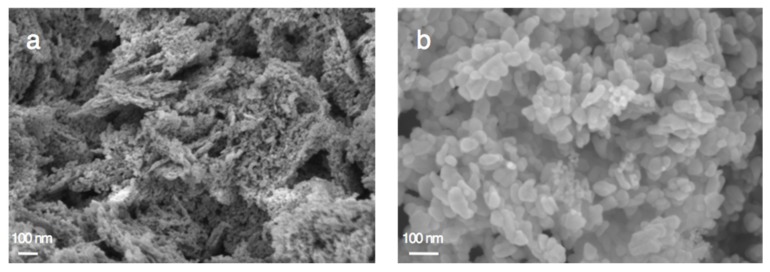
SEM images of (**a**) Cu_2_O-ZnO and (**b**) CuO-ZnO catalysts.

The main physico-chemical properties of the Cu_x_O/ZnO materials are reported in Table 1. It evidences that the synthesis of the CuO-ZnO and Cu_2_O-ZnO composite materials by photodeposition resulted in specific surface area and pore volume changes, and the composite materials displayed specific surface areas of 33 m^2^/g and 95 m^2^/g, respectively, with pore volumes of 0.24 cm^3^/g and 0.68 cm^3^/g. Figure 11 shows that the photon-assisted synthesis of Cu_x_O did not modify either isotherm or hysteresis types of type II or H3, respectively, while it slightly influenced the pore size distribution profiles. In addition, the SEM images shown in Figure 12 indicate that the overall morphology of the ZnO-based materials was not modified by the photon-assisted synthesis process.

**Figure 13:**
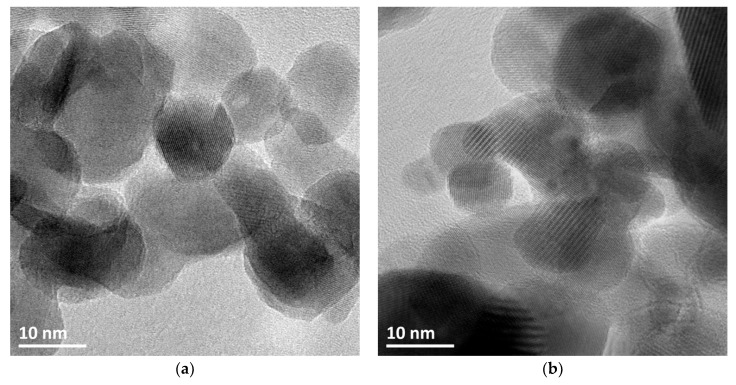
TEM images of the Cu-ZnO materials synthesized by photodeposition using (**a**) Cu(acac)_2_ and (**b**) Cu(NO_3_)_2_ as metallic precursor. An example of STEM imaging is shown as Figure S2.

Figure 13 shows the TEM images of the Cu-ZnO materials obtained by the photon-assisted synthesis method with both copper precursors. Unfortunately, the similarities of the interplanar spacings corresponding to the main planes of the ZnO material and of both Cu^(II)^O and Cu_2_^(I)^O crystallites did not allow the specific identification and location of the different phases within the Cu-ZnO materials that consisted of very entangled oxide crystallites in close contact. Indeed, the (101) and (100) planes of ZnO have interplanar distances of 0.25 nm and 0.28 nm (JCPDS No.00-036-1451), while the (111) and (002) planes of the Cu^(II)^O phase have 0.23 nm and 0.25 nm interplanar distances, respectively (JCPDS No. 01-089-5895) [41], and the (111) planes of the Cu_2_^(I)^O crystallites have an interplanar distance of 0.25 nm (JCPDS No. 00-005-0667) [42]. However, the presence of copper has been confirmed by performing overall EDS analysis on both samples, with a Cu content of 7.4 wt % and 9.5 wt % for the CuO-ZnO and the Cu_2_O-ZnO materials, respectively, in good agreement with the ICP-OES data.

It is worth noting that irrespective of the copper precursor, the prepared material can be considered as a Cu-ZnO composite catalyst, since both Cu_2_O and CuO crystallites do not exhibit significantly smaller mean sizes than the ZnO crystallites, rather than as a ZnO supported Cu catalyst that would usually consist of smaller size Cu-based nanoparticles dispersed on the ZnO support. Considering the exclusive presence of one single chemical state for the crystalline Cu-based crystallites in the samples as shown by XRD—keeping in mind the detection limit of the XRD measurement—the Cu-ZnO materials can thus be considered as Cu^(II)^O-ZnO and Cu_2_^(I)^O-ZnO composite catalysts, although the assignment of both transition metal oxide phases to specific crystallites in TEM images was not possible.

In addition, we cannot rule out that in addition to larger crystallites, some Cu species might remain highly dispersed on ZnO, thanks to a strong interaction between Cu and the amphoteric ZnO material that seems to protect the Cu nanoparticles from sintering, consequently providing enhanced interphase contact [43].

## 4. Conclusions

Cu_x_O-ZnO composite catalysts with a control of the chemical state of the copper oxide phase were prepared at room temperature using the solar light-induced redox photoactivity of the ZnO semiconductor support. The preparation of Cu_2_^(I)^O-ZnO and Cu^(II)^O-ZnO composite catalysts was achieved using Cu(acac)_2_ in THF-water and Cu(NO_3_)_2_ in water as metallic precursor, respectively.

The photoactive ZnO host material was prepared through the precipitation method with carbonate as precipitation agent, and its superior activity compared to ZnO synthesized by other methods was evaluated by taking the photocatalytic degradation of the 4-chlorophenol compound in water under simulated solar light as a model reaction.

Research is ongoing for understanding the key parameters driving the selective synthesis of the Cu_x_O phase within the ZnO-based catalysts. The activity of Cu_x_O-ZnO catalysts will be investigated in high interest reactions in the field of biomass conversion. Besides thermal catalysis applications, this work has opened a new route for the facile synthesis of Cu_2_O-ZnO heterojunction photocatalysts, which could take advantage under solar light of the heterojunction built between the *p*-type semi-conductor Cu_2_O with direct band gap of about 2.17 eV and the ZnO semiconductor phase.

## Figures and Tables

**Table 1:** Main physico-chemical properties of ZnO materials.

Sample	Mean Crystallite Size (nm) ^a^	BET Surface Area (m^2^/g)	Pore Volume (cm^3^/g)	Average Pore Diameter (nm)
ZnO-P-15	7	78	0.13	5
ZnO-P-60	11	66	0.09	6
ZnO-C-300	10	68	0.45	21
ZnO-C-350	22	41	0.24	21
ZnO-C-400	20	29	0.21	27
ZnO-C-500	18	29	0.24	30
ZnO-c-400	28	12	0.10	30
ZnO-c-500	30	6	0.05	28
Cu_2_O-ZnO	13	95	0.68	26
CuO-ZnO	20	33	0.24	29

^a^ derived from XRD pattern, as the mean size of the coherently-diffracting domains, derived from the Scherrer equation using the classical assumption of spherical crystallites. The full-width at half-maximum of the diffraction peaks of ZnO (102), (110), and (103) planes was used for the estimation.

**Table 2:** Pseudo first-order kinetic rate constant of 4-Cl-Phenol removal and zero order rate constant of TOC removal obtained on UV-A light cleaned ZnO catalysts (with the corresponding linear regression coefficients).

Catalyst	k’_TOC_ (mg/Lmin)/R^2^	k’_Phenol_ (min^−1^)/R^2^
ZnO-P15	0.097/0.99	0.008/0.98
ZnO-P60	0.130/0.88	0.011/0.94
ZnO-C300	0.180/0.99	0.210/0.99
ZnO-C350	0.173/0.90	0.017/0.99
ZnO-C400	0.180/0.99	0.014/0.99
ZnO-C500	0.071/0.99	0.007/0.97
ZnO-c400	0.030/0.89	0.010/0.93
ZnO-c500	0.153/0.98	0.007/0.98

**Table 3:** TOC conversion after several sequential photocatalytic runs with the ZnO-C300 catalyst.

Run	X_TOC_ (%)
First	100
Second	88
Third	61

## Supplementary Materials

The following are available online at http://www.mdpi.com/1996-1944/11/11/2260/s1. Figure S1: (a) Absorbance spectra of selected ZnO and Cu_x_O-ZnO photocatalysts and (b) the corresponding (αhυ)^1/2^ vs. hυ plots used to estimate the band gap of the ZnO materials using the K-M function *F*(*R*). Figure S2: Mapping STEM imaging recorded on CuO-ZnO composite material prepared from the Cu nitrate precursor: (red) Zn K, (blue) Cu K, (green) O K and (overlay).
