# Supplementary material for: Photoactive ZnO Materials for Solar Light-Induced CuxO-ZnO Catalyst Preparation
Source: Materials (Basel). 2018 Nov 13;11(11):2260. doi: 10.3390/ma11112260 (PMC6266916; doi:10.3390/ma11112260)
Supplement: Supplementary file 1 [file materials-11-02260-s001.pdf]

materials

*Supporting information*

# Photoactive ZnO materials for solar light induced $Cu_xO$-ZnO catalyst preparation

**Magdalena Brzezińska[1,2], Patricia García-Muñoz[2], Agnieszka M. Ruppert[1], and Nicolas Keller[2,*]**

[1] Institute of General and Ecological Chemistry, Faculty of Chemistry, Lodz University of Technology, ul. Żeromskiego 116, 90-924, Łódź, Poland ; agnieszka.ruppert@p.lodz.pl
[2] Institut de Chimie et Procédés pour l'Energie, l'Environnement et la Santé, CNRS/University of Strasbourg, 25 rue Becquerel, 67087 Strasbourg, France ; nkeller@unistra.fr
* Correspondence: nkeller@unistra.fr; Tel.: +33-3-6885-2811

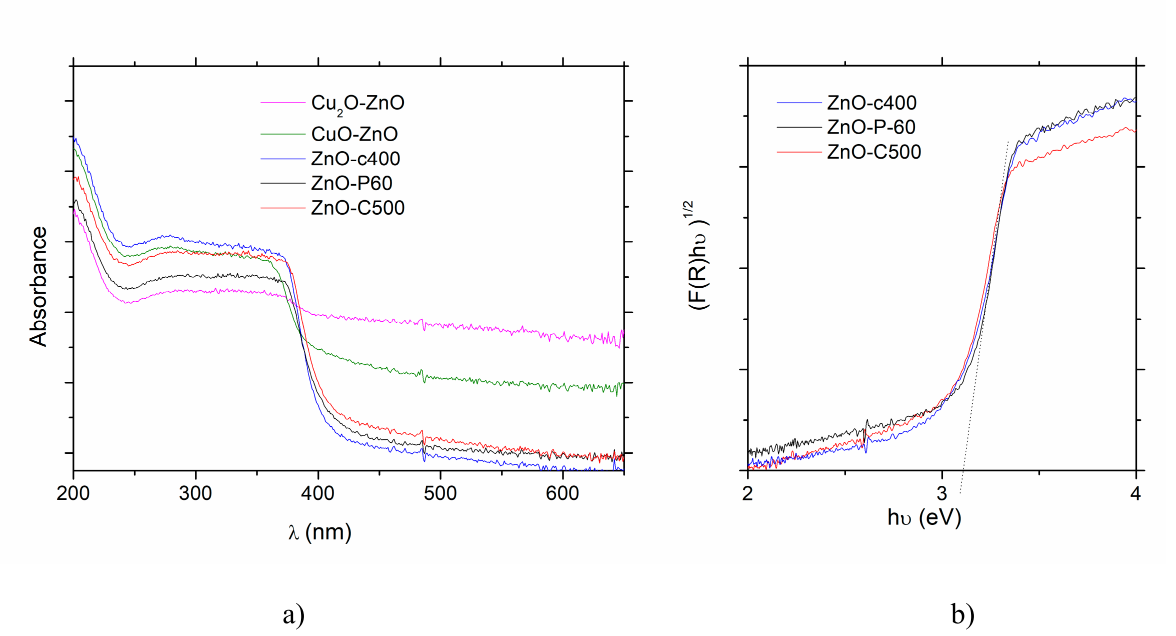


**Figure S1**. Absorbance spectra of selected ZnO and $Cu_xO$-ZnO photocatalysts (a) and the corresponding $(\alpha h\upsilon)^{1/2}$ *vs* hυ plots used to estimate the band gap of the ZnO materials using the K-M function *F(R)* (b).

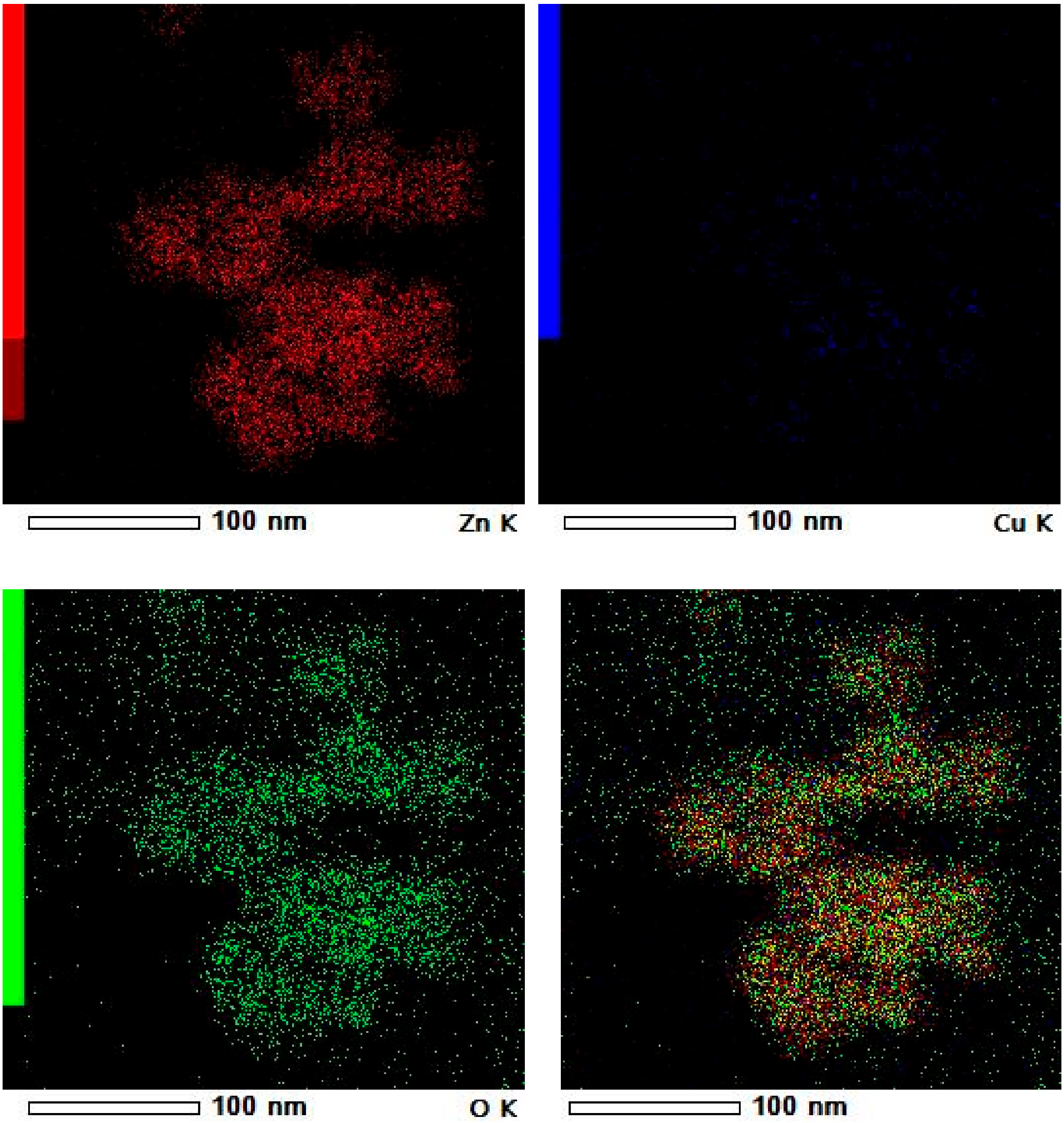


**Figure S2.** Mapping STEM imaging recorded on CuO-ZnO composite material prepared from the Cu nitrate precursor : (red) Zn K, (blue) Cu K, (green) O K and (overlay).
